# Supplementary material for: Modification of Polymeric Carbon Nitride with Au–CeO2 Hybrids to Improve Photocatalytic Activity for Hydrogen Evolution
Source: Molecules. 2022 Nov 3;27(21):7489. doi: 10.3390/molecules27217489 (PMC9656339; doi:10.3390/molecules27217489)
Supplement: Supplementary file 1 [file molecules-27-07489-s001.zip › molecules-1987067-supplementary.pdf]

Supporting Information

Modification of Polymeric Carbon Nitride with Au/CeO_2 Hybrids to Improve Photocatalytic Activity for Hydrogen Evolution

Linzhu Zhang [a,b], Lu Chen [a,b], Yuzhou Xia [a,b], Zhiyu Liang [a,b], Renkun Huang [a,b], Ruowen Liang [a,b*] and Guiyang Yan [a,b*]

[a] Department of chemistry, Fujian province university key laboratory of green energy and environment catalysis, Ningde Normal University, Ningde 352100, P. R. China

[b] State key laboratory of photocatalysis on energy and environment, Ningde Normal University, Ningde 352100, P. R. China

* Corresponding author: Prof. Ruowen Liang

Tel: +86-593-296427; E-mail: t1629@ndnu.edu.cn

* Corresponding author: Prof. Guiyang Yan

Tel: +86-593-2565503; E-mail: ygyfjnu@163.com

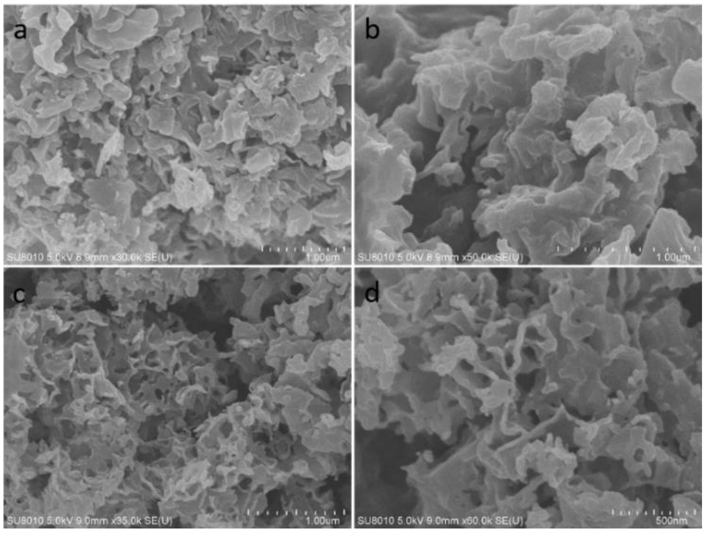

Figure S1. SEM images of (a), (b) CN, and (c), (d) 1.0% CeAu-CN.

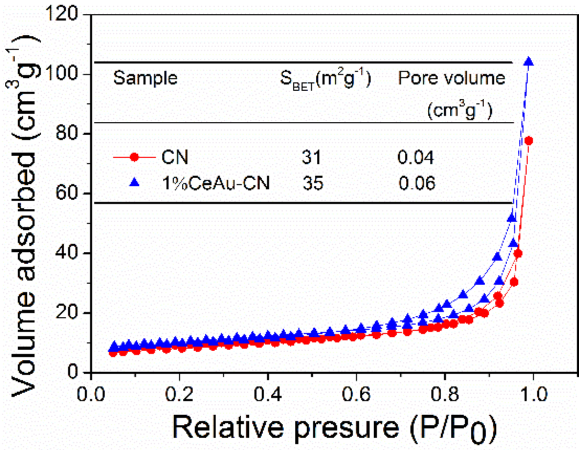

Figure S2. Nitrogen adsorption-desorption isotherms of CN and 1.0%CeAu-CN samples at 77K.

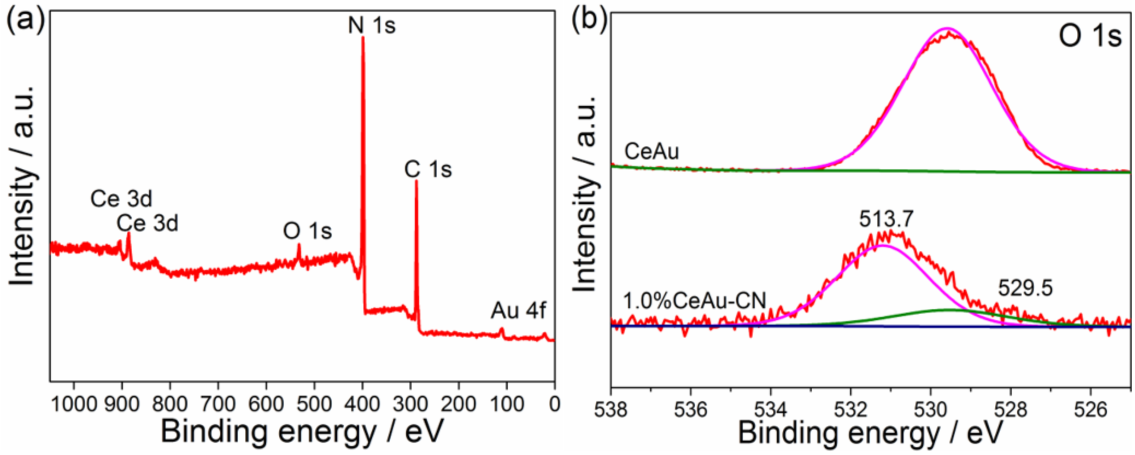

Figure S3. (a) Typical XPS survey spectra of 1.0%CeAu-CN and high resolution spectra of (b) O 1s of CeAu and 1.0%CeAu-CN.

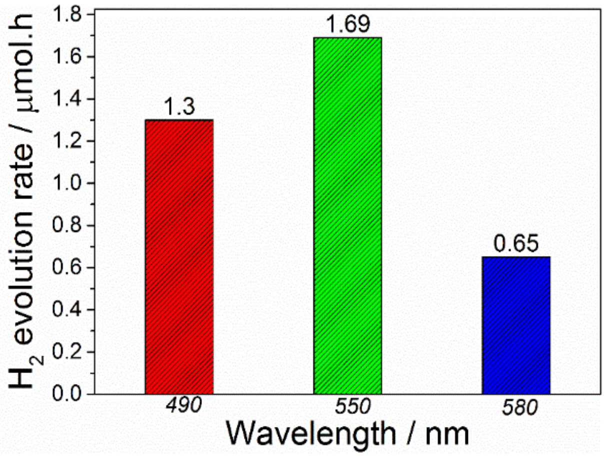

Figure S4. Photocatalytic activity for H_2 evolution rate of 1.0%CeAu-CN under different wavelength irradiation.

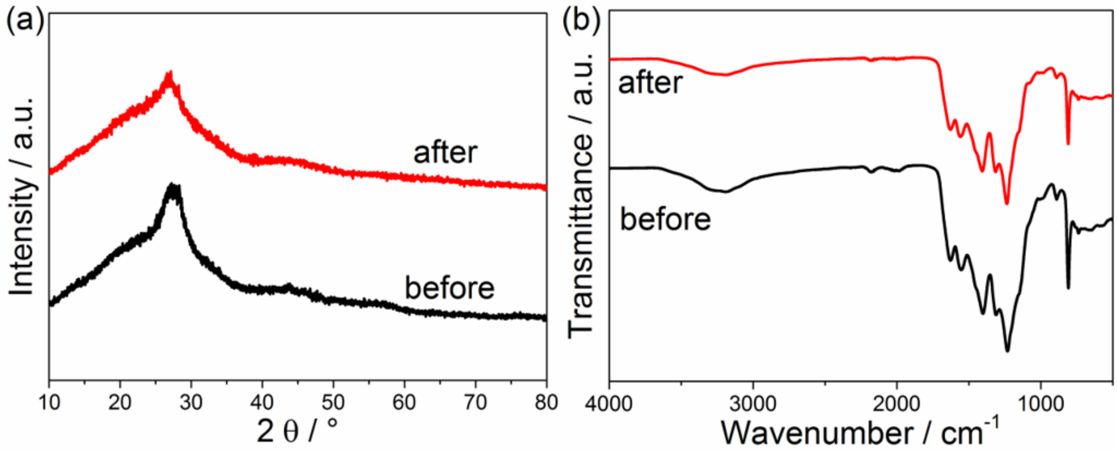

Figure S5. (a) XRD patterns and (b) FTIR spectra of 1.0%CeAu-CN samples before and after photochemical reaction.

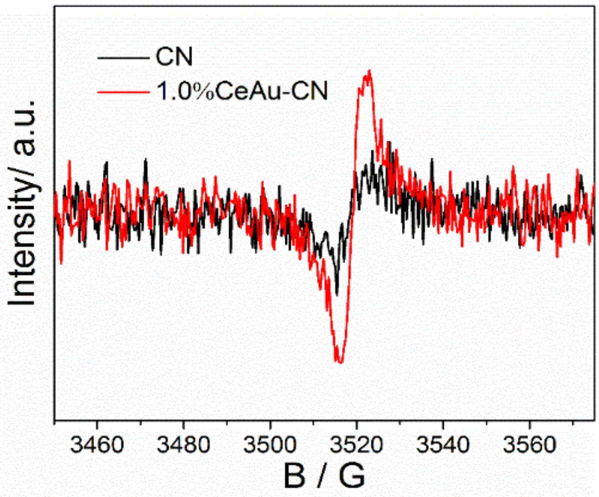

Figure S6. Room-temperature EPR spectra of CN and 1.0%CeAu-CN.

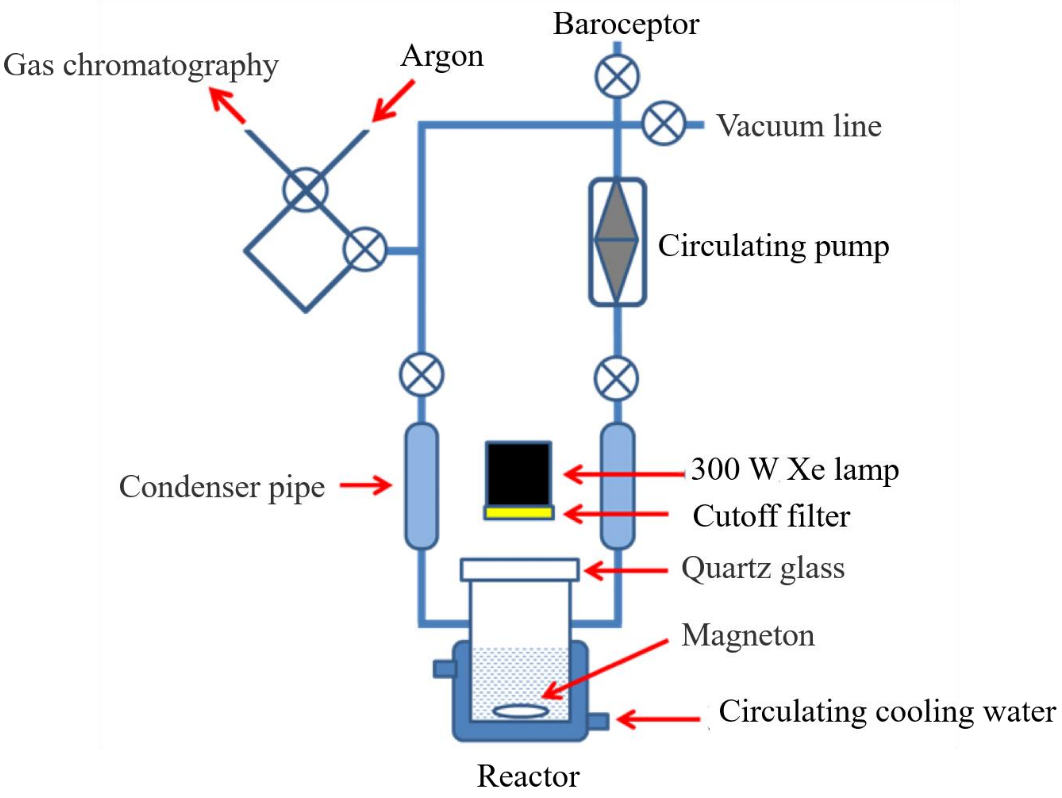

Figure S7. Schematic of photocatalytic water splitting system.
